# The Winding Road to Relapse: Forging a New Understanding of Cue-Induced Reinstatement Models and Their Associated Neural Mechanisms

**DOI:** 10.3389/fnbeh.2018.00017

**Published:** 2018-02-09

**Authors:** Mark D. Namba, Seven E. Tomek, M. Foster Olive, Joshua S. Beckmann, Cassandra D. Gipson

**Affiliations:** ^1^Department of Psychology, Arizona State University, Tempe, AZ, United States; ^2^Department of Psychology, University of Kentucky, Lexington, KY, United States

**Keywords:** discriminative stimulus, conditioned stimulus, relapse, neurobiology, reinstatement, addiction

## Abstract

In drug addiction, cues previously associated with drug use can produce craving and frequently trigger the resumption of drug taking in individuals vulnerable to relapse. Environmental stimuli associated with drugs or natural reinforcers can become reliably conditioned to increase behavior that was previously reinforced. In preclinical models of addiction, these cues enhance both drug self-administration and reinstatement of drug seeking. In this review, we will dissociate the roles of conditioned stimuli as reinforcers from their modulatory or discriminative functions in producing drug-seeking behavior. As well, we will examine possible differences in neurobiological encoding underlying these functional differences. Specifically, we will discuss how models of drug addiction and relapse should more systematically evaluate these different types of stimuli to better understand the neurobiology underlying craving and relapse. In this way, behavioral and pharmacotherapeutic interventions may be better tailored to promote drug use cessation outcomes and long-term abstinence.

## Introduction

Relapse triggered by cues associated with drugs of abuse is a hallmark of addiction and is a primary contributor to impeding success in maintaining abstinence. Discrete and contextual stimuli associated with previous drug use can initiate craving in individuals with substance dependence, potentially leading to subsequent relapse (Childress et al., 1993). In fact, it has been hypothesized that compulsive drug use could be a form of automatized behavior that is stereotyped, challenging to regulate, occurs separate from awareness, and is bound to stimuli associated with the drug (Tiffany and Carter, 1998). Individuals become sensitive to environmental stimuli that acquire motivational salience through repeated pairings with primary reinforcers, such as the presentation of food or drug (Bouton et al., 1993; O'Brien, 2003; Kalivas and Volkow, 2005; Weiss, 2010). Animal studies have established that these cues enhance drug self-administration, illustrating the importance of cues in maintaining drug use (Caggiula et al., 2001; Schenk and Partridge, 2001). These environmental stimuli become conditioned with the primary reinforcing effects of abused drugs, as can discriminative stimuli that predict drug presentation, both of which consistently produce drug seeking in animal models of cue-induced relapse (See, 2002; Shaham et al., 2003; Crombag et al., 2008; Bossert et al., 2013). Interestingly, cocaine-related stimuli (presented non-contingently) can evoke drug conditioned responses in cocaine-dependent humans (Ehrman et al., 1992).

Contingent, discrete environmental cues associated with drugs of abuse can produce increases in behavior previously reinforced by the drug when presented to an animal under conditions of abstinence. Additionally, physical environments contain a constellation of stimuli that can become occasion setters (OSs) and modulate the response-eliciting efficacy of discrete Pavlovian conditioned stimuli paired with drug self-administration (Gerber and Stretch, 1975). Likewise, these cues can serve a discriminatory function that predicts the availability of a drug of abuse upon the completion of a particular emitted response (Weiss et al., 2001). In addition to these salient, motivating cues or contexts, reinstatement of drug seeking (typically defined as increased responding on a previously drug-paired operandum following extinction) can be produced following presentation of other stimuli, including footshock or pharmacological stressors such as yohimbine (Erb et al., 1996; Buczek et al., 1999; Shaham et al., 2000) or priming injections of the previously self-administered drug (Gerber and Stretch, 1975).

Although the above preclinical models are frequently used to examine the neurobiology of drug relapse, it is important to critically examine their predictive validity (Epstein et al., 2006). It has been proposed that the process involved in behavioral change is unstable, thus lapse and relapse are likely (Bouton, 2014). From a learning perspective, changing behavior (i.e., inhibiting relapse) can be difficult since learning a new behavior does not erase the old behavior that is dependent on the context in which it is learned (Bouton, 2014; Bouton and Todd, 2014). Thus, lapse and relapse are especially probable if extinction of the drug-related behavior does not occur in the context in which drugs were taken. With that caveat, the reinstatement model of drug relapse has been widely used to examine the neurobiology underpinning lapse and relapse, and has been used to characterize potential vulnerabilities in the propensity to relapse (Knackstedt and Kalivas, 2009; Robinson et al., 2014).

In this review, we will examine the neurobiology underlying the process of encoding drug-related cues and how these cues might be encoded differentially depending on how they modulate behavior. As well, we will explore the neurobehavioral mechanisms underlying differential stimulus control of cues in motivated behaviors. Finally, we will discuss the importance of systematically examining and describing different types of cues in the discovery of neurobiological underpinnings of motivated behavior.

### Role of cues in drug-seeking behavior

Stimuli that are consistently paired with reward or reinforcers can come to influence reward-related behavior on their own, including drugs of abuse. These stimulus-reward relations are most obvious in Pavlovian paradigms, where a stimulus reliably predicts the occurrence of a food or drug reward. In addition to Pavlovian control, stimuli can also serve a discriminative or modulatory function, informing the organism as to the availability of a reward. Specifically, these stimuli inform an organism when a particular action will result in a specific reward (i.e., discriminative stimulus; S^D^; (Shahan, 2010), or when a specific stimulus will reliably predict a particular reward (occasion setter; OS; Holland, 1998); these modulatory relationships can also come to guide reward-related behavior on their own. Intriguingly, it appears that the neurobehavioral mechanisms engaged by a stimulus are dependent upon what function it serves (reinforcing or modulatory; Willuhn et al., 2010; Beckmann and Chow, 2015). Thus, understanding the role of stimulus-reward relationships in substance abuse and other reward-associated pathologies first requires an understanding of the neurobehavioral mechanisms involved in the different functional relationships between reward-associated stimuli and behavior. Above all, the dissociation of the neurobehavioral mechanisms underlying differential stimulus control might reveal novel, more-specific neurobehavioral targets to treat reward-associated pathologies, especially substance use disorders.

#### Control of drug seeking by contingent pavlovian conditioned stimuli

When an initially neutral stimulus is contiguously and consistently paired with a primary reward, like food or an abused drug, that stimulus can come to elicit responses on its own that are related to the primary reward it has been paired with, becoming a conditioned stimulus (CS). In preclinical models, one way experimenters utilize this process to study the role of stimulus control in drug-related behavior is to pair a neutral stimulus with an intravenous infusion of a drug of abuse; this is typically done by presenting the stimulus simultaneously with the drug infusion following an operant response (e.g., lever press). The repeated pairing with the primary (rewarding) effects of the drug transforms the stimulus into a drug-associated CS that can maintain the lever press alone.

Behavioral control via a CS is typically exhibited by response contingent CS-induced reinstatement of lever pressing following some extended period of extinction. Specifically, following the training period, the experimenter places the animal under extinction conditions, where lever presses no longer lead to the drug infusion or the associated CS. Over time, lever press behavior will decrease under extinction conditions. The experimenter will then present the CS alone following each lever press during reinstatement test sessions. The control of lever pressing via the CS is exhibited by the once extinguished operant response (lever press) being reinstated through the response contingent presentation of the drug-associated CS alone. It should also be noted that “incubation of craving” is another model in which contingent CSs maintain lever pressing alone. In this model, the animal undergoes forced abstinence for a period of time. When placed back into the drug-associated context (i.e., operant chamber), animals will emit lever presses under control of a contingent CS alone. Generally speaking, the amount of emitted lever pressing increases as more time elapses in forced abstinence (Conrad et al., 2008), hence the “incubation” of CS efficacy to maintain operant lever presses alone.

Importantly, the presentation of the CS alone during a reinstatement test session is contingent upon the previously trained lever-press behavior. Thus, from a behavioral perspective, the mechanism responsible for the reinstatement of the previously extinguished lever pressing is the conditioned reinforcing effects of the drug-associated CS. Through the repeated pairings between the CS and drug during self-administration, the CS comes to acquire reinforcing properties capable of maintaining lever pressing on its own. Although, it is important to note that these properties are short-lived without maintaining the drug-CS association. In typical contingent cue-induced reinstatement paradigms, lack of reconditioning to the CS results in an extinction of CS-related conditioned reinforcement with repeated reinstatement test sessions.

The conditioned reinforcing efficacy of drug-associated CSs is the most commonly used method for studying stimulus control of drug-seeking behavior (Bossert et al., 2013) in preclinical models and has demonstrated to be an effective method of study for many drug classes, including stimulants (Gipson et al., 2013a; Parsegian and See, 2014) and opiates (Bossert et al., 2005). Additionally, as stated above, the CS model has been extended to study long-term control of drug-associated stimuli following long periods of forced abstinence. Specifically, these studies have shown that the drug-associated CS can maintain greater rates of discrete cue-reinforced responding following longer periods of forced abstinence (Tran-Nguyen et al., 1998; Pickens et al., 2011). However, because of its contingent nature, it is not likely to model all types of stimulus control that individuals with substance use disorders are subjected to, as substance abusers are often exposed to drug-associated cues through non-contingent means.

#### Control of drug seeking by non-contingent stimuli

Non-contingent stimuli often take on a modulatory function, as opposed to the reinforcing function assumed by discrete Pavlovian CSs; they can serve to either inform the organism when a Pavlovian CS will effectively predict a future reward or when a particular action will result in a specific consequence. Despite the modulatory nature of these cues, they can have powerful control over drug seeking. First, it has been shown that context can serve as an OS, modulating the reinforcing efficacy of a drug-associated CS (Crombag and Shaham, 2002; Crombag et al., 2008). Following training of self-administration with a drug-CS relationship within a particular context (A), animals are then placed into a different context and responses then lead only to the CS alone (B). After responses for the CS alone have extinguished, animals are then placed back into the original training context (A), where responses again are followed only by the CS alone. This return to the original training context (A) after extinction in a different context (B) results in the renewal of responding for the CS.

Additionally it has been shown that environmental context can serve as a discriminative stimulus (S^D^). Specifically, the context in which an individual is placed can differentially signal the availability of drug following a particular operant response (Fuchs et al., 2007, 2008; Crombag et al., 2008; Lasseter et al., 2009, 2010, 2011, 2014; Ramirez et al., 2009). Following self-administration within a specific context (A), animals are then placed into a separate context (B) where responses result in no consequences. Following extinction of responding in the extinction context (B), animals are then returned to the original training context (A), where responding once again has no consequences. However, return to the original training context alone increases drug seeking responses. An S^D^ can also signal duration of access to drug self-administration and intake patterns can be brought under discriminative control. Specifically, the extended access model of self-administration (Ahmed and Koob, 1999), which is used to investigate a potential dysregulation of drug intake relevant to addiction in humans, has recently been found to be subject to stimulus control (Beckmann et al., 2012).

In addition to context, discrete S^D^s have been used to study stimulus control of drug seeking, where specific stimuli can be used to signal the availability of drug, the non-availability of drug, or the availability of a non-drug reinforcer (e.g., food; Weissenborn et al., 1995; Weiss et al., 2000, 2001, 2003; Stairs et al., 2010; McCuddy et al., 2014), all upon a specific operant response. This approach relies on the use of a multiple schedule, where changes in response-outcome contingency are split into components that are differentially signaled by a specific stimulus change throughout training. For example, left lever presses may lead to cocaine, but only in components coupled with the presence of a particular visual stimulus (e.g., left lever light), while right lever presses during this component are without consequence. Conversely, in the presence of a different stimulus (e.g., right lever light) during alternate components, right lever presses lead to food, while left lever presses within the alternate component are without consequence. Components typically alternate for fixed periods of time either within or across each training session. Following extinction of responses to either lever in the absence of either S^D^, non-contingent re-exposure to the previously trained S^D^s alone will specifically increase the response associated with that S^D^.

As discussed above, both CSs used as conditioned reinforcers and non-contingent exposure to S^D^s can reinstate drug-seeking behavior, although it appears they do so through different neurobehavioral processes. For instance, while contingent presentation of a drug-paired CS can reinstate drug seeking and produce alterations in accumbens synaptic plasticity, non-contingent presentation of the same CS does not (Grimm et al., 2000; Gipson et al., 2013a). These data support that reinstatement of drug seeking via CSs is operant and reinforced by the drug-paired CS. Contingent access to cocaine-associated S^D^s is much less robust than CSs, thus illustrating an important mechanistic difference between control of drug seeking through CSs and S^D^s (Di Ciano and Everitt, 2003). Notably, there are individual differences among rats in incentive salience attributed to discrete conditioned cues vs. non-contingent discriminative cues (Saunders and Robinson, 2010; Saunders et al., 2014). Specifically, discrete cocaine-associated cues increase drug seeking in rats that attribute motivational value to a discrete food cue, an effect that is absent in rats that do not attribute incentive salience to the food cue (Saunders and Robinson, 2010). Conversely, non-contingent S^D^s are less robust in renewing extinguished drug seeking in rats that attribute motivational value to a discrete food cue (Saunders et al., 2014). Taken together, these findings highlight the mechanistic differences between these varying stimulus modalities. Below we provide a detailed discussion of literature that suggests different neural mechanisms may underlie these distinct behavioral processes.

### Neurobiological differences in encoding conditioned vs. discriminative cues associated with natural and drug rewards

Understanding the neural mechanisms underlying stimulus control over drug-seeking behavior is critical to our understanding of drug addiction and relapse as a pathological disease state and developing effective treatment strategies to promote drug abstinence. In this section, we will first describe neuroadaptations within dopaminergic (DAergic) and glutamatergic systems found to underlie different types of cue-induced motivated behavior (from the current literature when possible). We will then describe drug-induced perturbations to other cellular and molecular mechanisms that are differentially expressed between conditioned and discriminative stimuli. Finally, we will highlight what future research is needed to examine novel targets that may underlie different types of cue-triggered motivated behavior, which may prove to be potentially effective pharmacotherapeutic avenues to improve drug use cessation outcomes. Tables 1, 2 provide a summary of neurobiological mechanisms underlying CS- and S^D^-induced reinstatement (respectively) across drug classes.

**Table 1:** Summary of neurobiological mechanisms underlying CS-induced reinstatement across drug classes.

**Experimental manipulation**	**Cocaine**	**Nicotine**	**Heroin**	**Alcohol**	**References**
Selective inactivation (baclofen + muscimol, tetrodotoxin, chemogenetics, or optogenetics)	NAcore↓, NAshell**–**, vHPC↓, PL↓, IL**–**, AC↓, BLA↓, LS → HPC↓, dAI↓	GI↓	NAcore↓, PL↓, IL↓, CeA↓, BLA↓, SN↓, VP↓	NAcore↓, NAshell**–**, PL↓, BLA↓	McLaughlin and See, 2003; Fuchs et al., 2004; Rogers and See, 2007; Rogers et al., 2008; Chaudhri et al., 2010, 2013; Forget et al., 2010; Gipson et al., 2013a; Stefanik and Kalivas, 2013; Cosme et al., 2015; Stefanik et al., 2016; Keistler et al., 2017; McGlinchey and Aston-Jones, 2017
D_1_ receptor antagonism	Systemic↓	Systemic↓	NAcore↓, NAshell**–**, PL↓		Alleweireldt et al., 2002; Bossert et al., 2007; See, 2009; Liu et al., 2010
D_1_ receptor agonism	Systemic↓, NAshell↑		Systemic↓^*^		Alleweireldt et al., 2002; Schmidt et al., 2006; Yue et al., 2014^*^
D_2_ receptor antagonism	Systemic↓	Systemic↓	Systemic↓^*^		Cervo et al., 2003; Liu et al., 2010; Yue et al., 2014^*^
D_2_ receptor agonism	NAshell↑	Systemic↓			Schmidt et al., 2006; Di Clemente et al., 2012
Restoration of EAAT-2/GLT-1	NAcore↓	NAcore↓	NAcore↓		Knackstedt et al., 2010; Trantham-Davidson et al., 2012; Shen et al., 2014b; Reissner et al., 2015
mGluR2/3 agonism	Systemic↓, NAcore↓	Systemic↓^‡^	Systemic↓	Systemic↓	Bossert et al., 2005; Moran et al., 2005; Peters and Kalivas, 2006; Zhao et al., 2006; Liechti et al., 2007^‡^; Smith et al., 2017
mGluR1/5 antagonism	Systemic↓, NAcore↓, dSTR**–**	Systemic↓		BLA↓, NAcore↓, Systemic↓	Bespalov et al., 2005; Adams et al., 2008; Schroeder et al., 2008; Kumaresan et al., 2009; Sinclair et al., 2012; Wang et al., 2013; Knackstedt et al., 2014; Smith et al., 2017
mGluR1/5 agonism	NAcore↑				Wang et al., 2013
GluN2A antagonism		NAcore↓			Gipson et al., 2013b
GlunN2B antagonism		Systemic↓	Systemic↓		Shen et al., 2011; Gipson et al., 2013b
AMPAR antagonism	NAcore↓, Systemic↓			Systemic↓	Bäckström and Hyytiä, 2004, 2006, 2007
MMP inhibition	NAcore↓	NAcore↓	NAcore↓		Smith et al., 2014
nNOS inhibition	NAcore↓				Smith et al., 2017
ECS activation	Systemic↑	Systemic↑	Systemic↑		De Vries et al., 2001, 2003; Gamaleddin et al., 2012
ECS inhibition	Systemic↓	Systemic↓	Systemic↓	Systemic↓	De Vries et al., 2001, 2003; De Vries and Schoffelmeer, 2005; Economidou et al., 2006; Ward et al., 2009

*When the experimental manipulation is applied either systemically or within a specific brain region, “↓” denotes decreased reinstatement, “↑” denotes increased reinstatement and “–” denotes no observed effect. “ → ” denotes a projecting pathway. Only studies employing extinction training prior to cue reinstatement are included here*.

* *Result of combined D_1_ agonism and D_2_ antagonism with L-Stepholidine*,

‡ *Also inhibited cue-induced food seeking. AC, anterior commissure; dAI, dorsal agranular insular area; BLA, basolateral amygdala; CeA, central amygdala; HPC, hippocampus; GI, granular insular cortex; IL, infralimbic cortex; LS, lateral septum; NAcore, nucleus accumbens core; NAshell, nucleus accumbens shell; PL, prelimbic cortex; SN, substantia nigra; vHPC, ventral hippocampus; VP, ventral pallidum*.

**Table 2:** Summary of neurobiological mechanisms underlying S^D^-induced reinstatement across drug classes.

**Experimental manipulation**	**Cocaine**	**Nicotine**	**Heroin**	**Alcohol**	**References**
Selective inactivation (baclofen + muscimol, tetrodotoxin, chemogenetics, or optogenetics)	OFC → BLA ↓, LS → HPC↓, dHPC → LS ↓, vHPC → LS**–**, BLA + dHPC↓, BLA + dmPFC↓, AI↓, SSJ**–**, AC↓		EC → dDG_ub_↓, vHPC → IL↓, vSub↓, vSub → NAshell, vmPFC → NAshell↓^#^	VP → VTA↓, VP → STN↓, BLA↓, PL↓, LH↓	**Fuchs et al.**, **2007****; Marchant et al.**, **2009****; Bossert et al.**, **2012**, **2016****; Chaudhri et al.**, **2013****; Torregrossa et al.**, **2013****; Bossert and Stern**, **2014****; Lasseter et al.**, **2014****; Prasad and McNally**, **2016****; Arguello et al.**, **2017****; Ge et al.**, **2017****; McGlinchey and Aston-Jones**, **2017****; Palombo et al.**, **2017****; Wang N. et al.**, **2017**
D_1_ receptor antagonism	Systemic↓,		NAcore**–**, NAshell↓, dlSTR↓, dmSTR**–**, vmPFC → NAshell↓^#^	Systemic↓, NAcore↓, NAshell↓	**Crombag et al.**, **2002****;** Liu and Weiss, 2002; **Bossert et al.**, **2007**, **2009**, **2012****; Chaudhri et al.**, **2009****; Lasseter et al.**, **2014****; Sciascia et al.**, **2014**
D_1_ receptor agonism	OFC↑			NAcore↓, NAshell↓	**Chaudhri et al.**, **2009****; Lasseter et al.**, **2014**
D_2_ receptor antagonism	Systemic↓			Systemic↓	**Crombag et al.**, **2002****;** Liu and Weiss, 2002
D_2_ receptor agonism					
Restoration of EAAT-2/GLT-1					
mGluR2/3 agonism	Systemic↓,		Systemic↓, NAshell↓, VTA↓		Baptista et al., 2004; **Bossert et al.**, **2004**, **2006****;** Cannella et al., 2013
mGluR1/5 antagonism	Systemic↓^‡^, NAcore↓, NAshell**–**, vCPu**–**				Martin-Fardon et al., 2009; **Xie et al.**, **2012**
mGluR1/5 agonism					
GluN2A antagonism					
GlunN2B antagonism					
AMPAR antagonism	NAcore↓, NAshell↓, vCPu**–**			BLA↓, NAshell**–**	**Millan and McNally**, **2011****; Xie et al.**, **2012****; Sciascia et al.**, **2015**
MMP inhibition					
nNOS inhibition				Systemic↓	Liu and Weiss, 2004
ECS activation					
ECS inhibition		Systemic↓			**Diergaarde et al.**, **2008**

*When the experimental manipulation is applied either systemically or within a specific brain region, “↓” denotes decreased reinstatement, “↑” denotes increased reinstatement and “–“ denotes no observed effect. “ → ” denotes a projecting pathway. Only studies employing extinction training prior to cue reinstatement are included here. Bolded references indicate the use of contextual S^D^s*.

# *Result of combined D1 antagonist in NAshell and B/M inactivation of vmPFC*.

‡ *Also inhibited cue-induced food seeking. AC, anterior commissure; Aid, agranular insular area; BLA, basolateral amygdala; CeA, central amygdala; vCPu, ventral caudate putamen; dDG_ub_, upper blade of dentate gyrus; EC, entorhinal cortex; dHPC, dorsal hippocampus; vHPC, ventral hippocampus; GI, granular insular cortex; IL, infralimbic cortex; LH, lateral hypothalamus; LS, lateral septum; NAcore, nucleus accumbens core; NAshell, nucleus accumbens shell; OFC, orbitofrontal cortex; PL, prelimbic cortex; dmPFC, dorsomedial prefrontal cortex; vmPFC, ventromedial prefrontal cortex; SSJ, somatosensory cortext; SN, substantia nigra; STN, subthalamic nucleus; dSTR, dorsal striatum; dlSTR, dorsolateral striatum; dmSTR, dorsomedial striatum; vSub, ventral subiculum; VP, ventral pallidum*.

#### Dopamine modulates drug-induced plasticity and drug seeking in a cue-specific manner

Cues that are conditionally paired with rewarding stimuli can alter patterns of neuronal activity, cell morphology, synaptic plasticity, and signal transduction. For instance, it is well known that natural reward-predicting CSs elicit responses in midbrain dopamine (DA) neurons such that neurons encode reward value and response pattern shifts from the occurrence of the reward itself to the CS associated with the reward (Waelti et al., 2001). Conditional and discriminative stimuli recruit distinct neurobiological mechanisms, where activation of certain neuronal subpopulations such as in the nucleus accumbens shell (NAshell) and core (NAcore) subcompartments, sustains motivated behavior that is dependent on stimulus function (Pezze et al., 2001; Ito et al., 2004; Kobayashi and Schultz, 2014).

**Figure 1:**
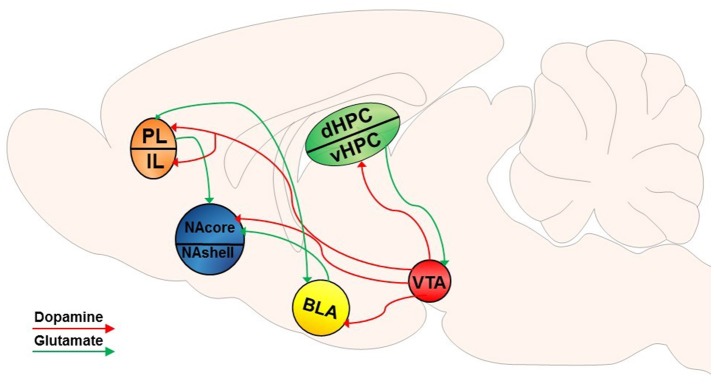
Dopamine-glutamate circuitry underlying CS-induced reinstatement of drug seeking. VTA dopamine (DA) input into the NAcore mediates CS-induced reinstatement. DA input into the PFC may also modulate rapid, transient glutamatergic plasticity in the NAcore in response to drug-associated CSs and mediates drug seeking behavior. DA input into the BLA may alter excitatory inputs into the NAcore and reciprocal BLA-PFC signaling. As well, DA input into the vHPC has been implicated in CS-induced reinstatement of drug seeking, and glutamatergic projections from the vHPC to the VTA may be an indirect pathway through which the HPC modulates dopaminergic input into other brain regions.

**Figure 2:**
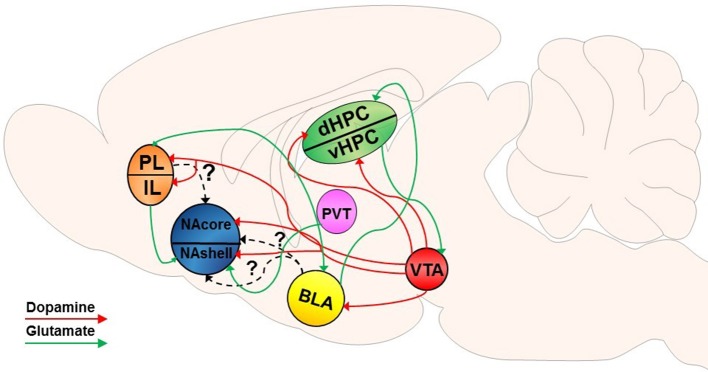
Dopamine-glutamate circuitry underlying S^D^-induced reinstatement of drug seeking. VTA dopamine (DA) input into the NAcore and NAshell mediates S^D^-induced reinstatement. DA input into the BLA and PFC have also been implicated in S^D^-induced reinstatement of drug seeking. Projections from the PFC to the NAshell have been implicated in S^D^-induced reinstatement; however, it is not known if PFC projections to the NAcore play a specific role in S^D^-induced reinstatement. Likewise, it is unclear if projections from the BLA directly to the NA play a role in S^D^-induced reinstatement akin to CS-induced reinstatement. The dHPC receives converging input from the BLA and VTA and has been implicated in S^D^-induced reinstatement. Similar to CS-induced reinstatement, the vHPC communicates bidirectionally with the VTA and may modulate dopaminergic input into the NA, PFC, and BLA. As well, glutamatergic projections from the PVT to the NAshell have also been shown to be involved in S^D^-induced drug seeking. Dotted lines indicate unknown effects of specific projecting pathways.

The NAcore is composed mainly of GABAergic medium spiny neurons (MSNs), accounting for 90–95% of the neuronal cell types within the striatum (Hedreen, 1981; Bolam, 1984; Chang and Kitai, 1985; Meredith, 1999). MSNs are typically segregated into one of two distinguishable populations, as they express either DA D_1_- or D_2_-like receptors (Gerfen et al., 1990; Gerfen and Surmeier, 2011). DA released from ventral tegmental area (VTA) efferents into the NA binds to these G-protein coupled receptors (GPCRs) on MSNs (Gerfen et al., 1987; Jimenez-Castellanos and Graybiel, 1987; Davidson and Stamford, 1993; Sesack et al., 2003). Although historically it was thought that D_1_ and D_2_-expressing MSNs were exclusively part of the direct and indirect basal ganglia pathways (Gerfen et al., 1990) and that manipulation of D_1_ or D_2_ MSNs specifically illustrates pathway specificity (Bock et al., 2013; MacAskill et al., 2014), recent studies have shown that this subdivision of cell type is not synonymous with these distinct pathways in projections of the NA (Smith et al., 2013; Kupchik et al., 2015; Kupchik and Kalivas, 2017). DAergic neurons in the VTA respond during conditioned behavior (Ljungberg et al., 1992) and, as described in detail below, conditioned and discriminative cues incorporate distinct DAergic mechanisms to modulate drug-induced neurobehavioral plasticity. Specifically, a vast body of literature has evaluated dopaminergic neuromodulation of drug-seeking behavior. Figures 1, 2 provide a non-exhaustive depiction of differential and overlapping glutamatergic and DAergic circuits found to be important in the induction of motivated drug seeking behavior by both types of cues. It should be noted that some projections may be involved in both types of motivated behavior, however these studies may not have been conducted yet to show overlapping (or differential) involvement.

##### Dopaminergic signaling in CS-induced reinstatement of drug seeking

A rich body of literature has shown that after repeated pairings of a stimulus with an appetitive reward, DA neurons in the pars compacta of the substantia nigra and VTA respond phasically to CSs rather than to the reward itself and are dependent on event unpredictability (Schultz et al., 1997; Hollerman and Schultz, 1998; Schultz, 1998). GPCRs are modulatory and historically have been thought to deliver general neuromodulation with relatively slow time resolution. Given the possible dichotomous role of neuromodulatory DA signaling in delivering both fast and slow-timed information to other systems (Schultz et al., 1997), it is likely that extracellular DA release during relapse delivers precisely timed information to specific structures altering the neural environment in a modulatory manner and impacting behavioral output. For example, DA signaling could deliver precise information to impact rapid, transient plasticity associated with drug seeking (described in detail below).

Recent studies have begun to further elucidate the role of NAcore DA release in the reinstatement of drug seeking as well as in the neurobiological changes that occur within the NAcore during reinstatement. For instance, Spencer et al. (2016) reported that re-administration of cocaine, which increases extracellular DA (Willuhn et al., 2010), reversed the rapid, transient plasticity associated with CS-induced cocaine seeking (involvement of rapid, transient glutamatergic plasticity during drug seeking is described in more detail below). Additionally, antagonism of VTA D_1_ (Alleweireldt et al., 2002) and D_2_ receptors has been shown to inhibit both reinstated cocaine seeking as well as rapid synaptic plasticity in the NAcore (Shen et al., 2014a). Another recent study discovered a positive correlation between prelimbic (PL)-NAcore pathway activation and CS-induced cocaine reinstatement (McGlinchey et al., 2016). This projection was found to be DA-dependent, where pharmacological inhibition of DA signaling in the PL attenuated discrete CS-induced cocaine reinstatement but not sucrose seeking in drug-naïve animals. This DAergic neuromodulation of prefrontal glutamate release into the NA is also supported by a previous study that found that DA efflux was only increased in the dorsomedial prefrontal cortex (dmPFC) and not in the NA during CS-induced, methamphetamine-induced, and CS + methamphetamine-induced reinstatement (Parsegian and See, 2014). Taken together, these results highlight the possibility that extracellular DA release may suppress NAcore glutamatergic plasticity associated with discrete CS-induced drug seeking. As well, these data further support the role of DA in modulating glutamatergic signaling during motivated behavior.

DAergic signaling may also serve to encode prediction errors to adjust reward-seeking behavior in response to new environmental contingencies (Hollerman and Schultz, 1998; Schultz, 1998). Fast-scan cyclic voltammetry (FSCV) studies, which use electrodes that emit fast, alternating electrical currents that rapidly oxidize and reduce an electroactive molecule of interest to monitor rapid fluctuations in extracellular levels, have shown that DA release can change rapidly and transiently in response to distinct conditioned stimuli that predict changes in reward. For instance, it has been demonstrated that a discrete CS that is paired with delayed cocaine availability is associated with blunted DA levels, which are then rapidly elevated when a discrete cue paired with immediate cocaine delivery is presented (Wheeler et al., 2011). Another study utilizing FSCV found that DA was released in the NA transiently in response to a discrete auditory cue paired with a food reward during appetitive Pavlovian conditioning. Extinction training significantly reduced DA release in response to the cue but was reinstated after two non-contingent presentations of the reward (Sunsay and Rebec, 2014). There is also evidence to suggest that mesolimbic DA signals are transduced differentially following the presentation of a reward-paired cue and directly following a goal-directed behavior (e.g., a lever press). For instance, one study found that DA-mediated neuronal responses to conditioned cues associated with intracranial stimulation of the VTA specifically recruited D_2_-expressing neurons in the NA, whereas DA-mediated responses that were temporally proximal to lever presses involved both D_1_- and D_2_-expressing neurons (Owesson-White et al., 2016). These studies demonstrate that DA neurotransmission and signal transduction can change rapidly and transiently in response to drug-associated CSs and following goal-directed behavior to modulate future reward-seeking behavior.

Research investigating the dichotomous functional roles of the NAcore and NAshell indicates that DA input into these two sub-regions depends on behavioral and environmental conditions. For example, non-contingent presentation of a discrete conditioned stimulus (i.e., a light) that was previously paired with an infusion of cocaine selectively increased DA into the NAcore, while DA levels were elevated in both the NAcore and NAshell following intravenous cocaine self-administration in rats (Ito et al., 2000). As mentioned previously, non-contingent exposure to drug-paired CSs does not sufficiently reinstate drug seeking or produce changes in synaptic plasticity within the NA. As such, the neuromodulatory influence of DA on neurobehavioral plasticity in response to drug-paired cues may be dependent on the contingency between the operant response and the CS together, as opposed to the CS itself. Specifically, the CS-induced surge of DA may essentially reinforce the behavioral response. Similar to the disparate roles of the NAcore and NAshell in CS-induced reinstatement, D_1_- and D_2_-type MSNs also appear to have distinct functional roles in mediating drug seeking. For instance, one recent study found that loss of GABAergic plasticity in D_2_-type (but not D_1_-type) MSNs projecting to the ventral pallidum (VP) potentiates CS-induced reinstatement of cocaine seeking (Heinsbroek et al., 2017). As well, we recently discovered that D_1_(+) rather than D_1_(–) MSNs mediate rapid, transient synaptic plasticity during CS-induced cocaine seeking within the NAcore (Bobadilla et al., 2017). Together, these studies highlight the differential roles of D_1_- vs. D_2_-type MSN signaling in CS-induced drug seeking.

##### Dopaminergic signaling in S^*D*^-induced reinstatement of drug seeking

Although DA has been shown to be important in S^D^-induced drug seeking behavior (e.g., alcohol, Sciascia et al., 2014; Marchant and Kaganovsky, 2015; cocaine, Lasseter et al., 2014; heroin, Bossert et al., 2009), it remains unclear if DA impacts rapid synaptic plasticity associated with S^D^-induced drug seeking. The only study to date examining rapid plasticity in S^D^-induced drug seeking did not examine DAergic modulation of this neural event (Stankeviciute et al., 2014). Unlike CS-induced reinstatement, studies utilizing S^D^-induced reinstatement of drug seeking following extinction training demonstrate that both the NAcore and NAshell are involved in S^D^-induced drug seeking (Fuchs et al., 2004, 2008; Chaudhri et al., 2009). However, one study has demonstrated that antagonism of D_1_ receptors in the NAshell, but not the NAcore, is sufficient to attenuate S^D^-induced reinstatement of heroin seeking (Bossert et al., 2007). While research on this topic is limited, it appears S^D^-induced drug seeking may not employ the same degree of DA receptor specificity as CS-induced reinstatement. Nevertheless, antagonism of DA receptors in the dorsal prefrontal cortex (dPFC), which receives DA input from the VTA and sends excitatory glutamatergic projections to the NA, was shown to inhibit cocaine-primed reinstatement, while DA release into the NAcore and ventral pallidum (VP) were not causally associated with drug-primed reinstatement (McFarland and Kalivas, 2001). Considering prefrontal glutamate release into the NA is required for cocaine-primed reinstatement of drug seeking (McFarland et al., 2003), it may be that DAergic stimulation of Gs-coupled D_1_ receptors in the PFC is necessary for activation of glutamatergic projections to the NA, a mechanism which may extend to S^D^-induced drug seeking (Sciascia et al., 2014).

##### Dopaminergic projections to other neural structures involved in CS- and S^*D*^-induced drug seeking

Beyond the NA, the functional roles of the amygdala (AMY) and the hippocampus (HPC) in reward seeking are a subject of intense study, as both regions communicate either directly or indirectly with the VTA and project to the NA. Importantly, previous studies have revealed their role in mediating cue-triggered behavioral responses. For instance, it has been demonstrated that selective inactivation of the dorsal hippocampus (dHPC), the basolateral amygdala (BLA), and the dmPFC abolishes contextual S^D^-induced cocaine reinstatement, whereas inactivation of the dHPC did not alter discrete CS- or drug-induced reinstatement (Fuchs et al., 2005). However, context by nature is a complex spatial array of stimuli, and thus use of a punctate stimulus could uncover differential involvement of various sub-regions of the HPC. Other studies suggest that the ventral hippocampus (vHPC) may also be involved in S^D^-induced (Lasseter et al., 2010) as well as CS- and drug-induced reinstatement (Rogers and See, 2007). The HPC-VTA loop has been characterized by several studies (see Lisman and Grace, 2005, for review) and is thought to contribute to long-term potentiation (LTP) and changes in synaptic plasticity required for learning and memory in goal-directed tasks (Wise, 2004). One study found that stimulation of the vHPC increased VTA DA neuron activity and NA DA levels, suggesting a neuromodulatory role of the vHPC on DAergic input into the NA (Legault et al., 2000). Curiously, another study found that vHPC activation increased DA levels in the NAshell and that dHPC activation had an inhibitory effect on extracellular DA levels in the NAcore (Peleg-Raibstein and Feldon, 2006), which might suggest that the NAcore is not involved in HPC-mediated activation of VTA DA release in S^D^-induced reinstatement.

In addition to the HPC, the BLA receives significant DAergic input and appears to be especially involved in mediating CS-induced reinstatement (Weiss et al., 2000; See et al., 2001; Kantak et al., 2002). Specifically, both D_1_- and D_2_-like receptors in the BLA appear to be involved in the formation of cocaine-CS associations that are required for CS-induced reinstatement of cocaine seeking (Berglind et al., 2006). As well, the BLA and PFC form strong reciprocal connections that have been shown to regulate contextual S^D^-induced reinstatement of cocaine seeking (Lasseter et al., 2011). As it appears, the functional heterogeneity of brain regions such as the NA, AMY, and HPC may be demonstrated with distinct cues that recruit specific and potentially differential neural pathways to promote the acquisition and maintenance of reward seeking as well as relapse vulnerability.

#### Glutamatergic plasticity mediates drug seeking and relapse vulnerability

Dysregulations in glutamate neurotransmission between key brain regions along the mesocorticolimbic pathway have been found to underlie the chronic, compulsive, relapsing use of drugs that characterizes addiction. Several studies have elucidated key glutamatergic pathways along the reward circuit that are recruited differentially depending on the type of relapse model being utilized. Projections from the PFC (prelimbic and infralimbic cortex; PL and IL, respectively), AMY, HPC, and thalamus (THAL) innervate the NAshell (Groenewegen et al., 1999). Importantly, projections from the BLA to the PL as well as the BLA to dHPC have been implicated in the neural circuit underlying contextual S^D^-induced cocaine seeking (Fuchs et al., 2007). The paraventricular thalamus (PVT) is important in contextual S^D^-induced drug seeking (James et al., 2011), and its projection to the NAshell is important in contextual S^D^-induced alcohol seeking (using decarbonated beer) (Hamlin et al., 2009). Additionally, glutamatergic projections from the PL to NAcore have been found to be involved in CS-induced cocaine reinstatement (Gipson et al., 2013a; Stefanik et al., 2016), whereas ventromedial PFC (vmPFC, encompassing the IL; Peters et al., 2013) to NAshell has been implicated in contextual S^D^-induced heroin seeking (Bossert et al., 2012). Recently, glutamatergic projections from the BLA to the NAcore has been found to be important in CS-induced cocaine seeking (Stefanik and Kalivas, 2013).

##### Glial glutamate transport critically mediates CS-induced reinstatement of drug seeking

Research investigating dysregulations in the molecular mechanisms driving glutamate signaling after chronic exposure to drugs of abuse has revealed that these aberrant neuroadaptations are dependent upon behavioral and environmental conditions. The glial glutamate transporter (GLT-1/EAAT-2), which is responsible for regulating >90% of extracellular levels of glutamate, is one key neural substrate that has been implicated in drug relapse. In addition, the sodium-independent cystine-glutamate exchanger, system xc- (also referred to by its catalytic subunit xCT), works synergistically with GLT-1 to provide glutamatergic tone on presynaptic mGluR2/3 autoreceptors to limit presynaptic release of glutamate (Moran et al., 2005; Kalivas, 2009). Particularly, GLT-1 function and expression has been shown to modulate the expression of CS-induced reinstatement. For example, one study demonstrated that the β-lactam antibiotic ceftriaxone attenuated CS-induced cocaine reinstatement and increased GLT-1 in the NAcore, but not the NAshell (Fischer et al., 2013). Ceftriaxone has been found to up-regulate xCT in addition to GLT-1 (Knackstedt et al., 2009, 2010; Alhaddad et al., 2014), restore basal levels of extracellular glutamate following cocaine self-administration (Trantham-Davidson et al., 2012), and is considered a potential therapeutic target for attenuating relapse in humans (Reissner and Kalivas, 2010). Similar studies have also shown that up-regulation of GLT-1 in the NA is associated with decreased CS-induced reinstatement (Sari et al., 2009; Knackstedt et al., 2010; Sondheimer and Knackstedt, 2011).

Akin to ceftriaxone, the cysteine prodrug *N*-acetylcysteine (NAC) is associated with an up-regulation of both xCT and GLT-1 (Knackstedt et al., 2010) and has previously been shown to inhibit cocaine-primed reinstatement (Baker et al., 2003; Moran et al., 2005) as well as both CS- and heroin-induced drug seeking (Zhou and Kalivas, 2008), CS-induced cocaine seeking (Reissner et al., 2015), and CS-induced nicotine seeking (Ramirez-Niño et al., 2013). It was recently discovered that chronic NAC treatment inhibits CS-induced cocaine reinstatement through a GLT-1-dependent mechanism, where inhibition of xCT expression did not block NAC's attenuating effect on reinstatement (Reissner et al., 2015). While NAC is believed to drive system xc- and depotentiate glutamatergic afferents in the NA, the pharmacotherapeutic potential and clinical relevance of specifically driving system xc- remains somewhat unclear. Although one study found that NAC-mediated attenuation of cocaine primed-reinstatement was reversed when system xc- was pharmacologically inhibited (Kau et al., 2008), it is still unclear whether driving system xc- is a prerequisite for NAC-mediated attenuation of CS-induced relapse and if this effect is drug-specific. Likely, there are other mechanisms underlying NAC's therapeutic potential that have yet to be fully elucidated.

##### Glial glutamate transport in S^*D*^-induced reinstatement of drug seeking

As mentioned above, up-regulation of GLT-1 in the NAcore but not the NAshell is associated with a decrease in CS-induced reinstatement of cocaine seeking (Fischer et al., 2013). One recent study that examined GLT-1 expression and glutamate efflux after a period of forced abstinence found that although ceftriaxone up-regulated GLT-1 and attenuated drug seeking, glutamate efflux was not reduced during cocaine seeking induced by drug context (LaCrosse et al., 2016). Thus, despite showing similar ameliorations to dysregulated glutamatergic signaling and subsequent behavior as described in (Trantham-Davidson et al., 2012) (which utilized a cocaine-induced reinstatement model), failure of ceftriaxone to inhibit glutamate efflux in this study potentially highlights a differential role of glial glutamate transport between different models of reinstated/renewed drug seeking. The role of glial-glutamate transport in S^D^-induced reinstatement of drug seeking is poorly understood and more research is needed to fully characterize the functional differences in glutamate efflux during reinstatement between drug-paired CSs and S^D^s.

##### Dendritic morphology and physiology at glutamatergic synapses alters in response to drug-associated CSs

Acute and chronic exposure to drugs of abuse is associated with changes in synaptic structure and function that increase sensitivity to drug-associated cues. Early studies using Golgi-cox staining to examine dendritic morphology in the PFC and NA revealed that repeated exposure to psychostimulants (Robinson and Kolb, 1997; Brown and Kolb, 2001) as well as morphine (Robinson and Kolb, 1999) is associated with alterations in dendritic complexity and increases in spine density. More recently, it has been demonstrated that nicotine self-administration and extinction training is associated with enduring increases in basal spine head diameter, increases in AMPA to NMDA excitatory post-synaptic currents (EPSCs), an increase in AMPA (GluA1), and NMDA (GluN2A and GluN2B) receptor subunit expression, and a decrease in GLT-1 expression within the NA (Gipson et al., 2013b). In this study, CS-induced reinstatement was also associated with rapid, transient plasticity, such as increases in extracellular glutamate, dendritic spine diameter, and AMPA to NMDA ratios within 15 min of cue exposure. Similar changes in transient synaptic plasticity have also been observed following CS-induced cocaine seeking, which requires glutamatergic input from the PL (Gipson et al., 2013a). Interestingly, another study examining the role of GluA1 AMPA receptor subunits in cocaine seeking found that targeted deletion of GRIA1 (i.e., the gene that encodes GluA1 AMPAR subunits) in mice was not associated with changes in cocaine self-administration or discrete CS-induced reinstatement (Mead et al., 2006).

Both acute and chronic cocaine exposure has been linked to increases in GluA2-lacking, calcium permeable AMPA receptors (CP-AMPARs), which is thought to underlie “incubated” CS-induced reinstatement of drug seeking (Conrad et al., 2008; Wolf, 2010). Additionally, early withdrawal from cocaine is associated with the presence of “silent synapses” between the BLA and NAcore, which are often characterized by the expression of stable NMDA receptors (NMDARs) and labile AMPARs that are inserted into the membrane after prolonged withdrawal (Lee et al., 2013). Incubation of drug craving is generally characterized by time-dependent increases in drug seeking and corticostriatal activity following an extended period of withdrawal (Tran-Nguyen et al., 1998; Pickens et al., 2011). However, incubation of drug craving is often measured as an increase in drug seeking in response to discrete conditioned cues in the same context in which the drug was initially administered. Importantly, it is unknown how S^D^s impact incubation of drug seeking behavior. Thus, understanding the differential role of S^D^-induced alterations in synaptic structure and function is necessary to fully elucidate the neurobiological underpinnings of complex drug-associated stimulus interactions.

In addition to studies utilizing CS-models, a necessary consideration must be made regarding drug-induced reinstatement models, as some of the neuroadaptations described herein between CS- and S^D^-models have also been observed similarly or differentially in drug-induced reinstatement paradigms. Acute cocaine exposure is associated with an increase in dendritic spine density and synaptic plasticity in the VTA (Sarti et al., 2007). In addition to changes in spine density, it has been demonstrated that withdrawal from chronic, non-contingent cocaine exposure induces changes in spine head diameter in the NAcore, as well as rapid alterations in dendritic morphology in response to a cocaine challenge similar to those observed in CS-induced reinstatement models (Shen et al., 2009). Another study examining AMPA to NMDA ratio and spine head diameter in the NAcore following cocaine-primed reinstatement found that inhibition of the PL potentiated AMPA to NMDA currents and spine head diameter, while inhibition of the VTA or administration of D_1_/D_2_ antagonists inhibited such changes (Shen et al., 2014a). Intriguingly, inhibition of the PL was still associated with a decrease in cocaine-primed reinstatement of drug seeking. This poses an interesting contrast to what has been observed previously in a CS-induced reinstatement model of cocaine seeking (Gipson et al., 2013a).

##### S^*D*^-induced alterations in dendritic structure and function at glutamatergic synapses

While the role of CP-AMPARs in S^D^-induced reinstatement has not been fully characterized, recent evidence suggests that a reduction in GluA1 expression in the NA may be associated with a decrease in contextual S^D^-induced cocaine seeking (LaCrosse et al., 2016). Given these findings, it appears that changes in dendritic spine structure, AMPA to NMDA ratios, and AMPAR expression in the NA may not be unconditionally linked to all forms of reinstated drug seeking. Rather, it appears that reinstatement may depend on stimulus function-specific neural mechanisms. While these changes in dendritic spine morphology and physiology have been examined extensively in discrete conditioned cue- and drug-induced reinstatement, very few studies have attempted to elucidate whether discriminative cues elicit similar cellular responses. However, one recent study demonstrated that re-exposure to a cocaine-associated environment renewed cocaine seeking following extinction training in a separate environment and produced similar alterations in dendritic spine head diameter as in the previously mentioned studies that utilized non-contingent cocaine injections or conditioned cues (Stankeviciute et al., 2014). Taken together, certain alterations in synaptic potentiation and dendritic spine morphology may serve as a common neurobiological mechanism underlying drug seeking across drug types and environmental conditions (Scofield et al., 2016). However, more work will need to be conducted to more clearly define the underlying cellular and molecular mechanisms that drive alterations in dendritic morphology and physiology across different behavioral conditions and in response to different cue types.

#### Future neurobiological targets underlying motivated behavior

##### Brain- and glial cell line-derived neurotrophic factors

In addition to DAergic and glutamatergic signaling, drug-induced alterations in synaptic plasticity and signal transduction along the mesocorticolimbic pathway are mediated by neurotrophic factors, neuropeptides, extracellular matrix substrates, and other signaling molecules. Brain-derived neurotrophic factor (BDNF) is one neurotrophic factor that has been extensively studied and is known to produce enduring neuroadaptations in response to drugs of abuse. For instance, BDNF increases progressively over time in response to cocaine (but not sucrose) withdrawal in the NA, VTA, and AMY, which is also thought to underlie incubation of drug craving as described previously (Grimm et al., 2003). The mPFC is the primary source of striatal BDNF and has been demonstrated to mediate cocaine seeking such that acute administration of BDNF into the mPFC produces time-dependent attenuation of CS-induced cocaine seeking following self-administration with no effect on food-seeking behavior (Berglind et al., 2007, 2009). As well, BDNF expression and its effects on drug seeking appears to be dependent on withdrawal, where elevated levels in the NAcore and NAshell following extended cocaine self-administration are detectable on withdrawal day (WD) 45 and WD90, respectively (Li et al., 2013). Similar to BDNF, glial cell line-derived neurotrophic factor (GDNF) expression in the VTA is associated with time-dependent increases in CS-induced cocaine seeking following a period of withdrawal (Ghitza et al., 2010). As well, heroin self-administration and withdrawal is associated with time-dependent increases in GDNF mRNA (but not protein levels) in the VTA and NA, and exogenous administration of GDNF into the NA is associated with increased CS-induced heroin seeking (Airavaara et al., 2011). Administration of GDNF into the VTA is also associated with an increase in extinction responding following cocaine self-administration (Lu et al., 2009). Considering the effects of BDNF and GDNF are drug-specific, brain region-specific, and time-dependent, it is unlikely that a systemic pharmacotherapeutic targeting some aspect of BDNF or GDNF signal transduction would be clinically efficacious (Ghitza et al., 2010). Regardless, understanding the heterogeneous profile of expression of neuromodulators such as BDNF and GDNF is necessary to more clearly elucidate differential neural mechanisms governing drug seeking.

##### Neuropeptides

Neuropeptides originating from the lateral hypothalamus (LH) such as orexin (i.e., hypocretin) and melanin-concentrating hormone (MCH) have long since been implicated in regulating feeding behavior (DiLeone et al., 2003) and may also drive drug seeking. For instance, MCH signaling from the LH to the NA may underlie the rewarding aspects of feeding (Saper et al., 2002) and MCH signaling has also been shown to sensitize animals to the rewarding effects of psychostimulants (Cabeza de Vaca et al., 2002). A recent study found that the LH has glutamatergic and GABAergic projections that innervate both dopaminergic and GABAergic neurons in the VTA (Nieh et al., 2015). The LH also sends glutamatergic projections to the lateral habenula (LHb), which projects to VTA/rostromedial tegmental nucleus (RMTg, or tail of the VTA, tVTA; Jhou et al., 2009; Kaufling et al., 2009) GABAergic neurons that are capable of inhibiting VTA dopaminergic neurons (Poller et al., 2013). Electrical stimulation of the LHb is associated with decreases in both cocaine self-administration and extinction responding (Friedman et al., 2010) and also mediates the aversive effects of nicotine (Fowler and Kenny, 2014). Conversely, exposure to discrete heroin CSs and reinstated heroin seeking is associated with an increase in c-Fos expression in the medial portion of the LHb (Zhang et al., 2005). Currently, there is little evidence that clearly distinguishes the role of the LHb in CS- vs. S^D^-induced reinstatement. Similar to MCH, orexin neurons in the LH project to multiple corticolimbic structures (Peyron et al., 1998) and have been implicated in mediating reward-seeking behavior (Aston-Jones et al., 2010). Antagonism of OxR1 (but not OxR2) inhibits CS-induced cocaine reinstatement (Smith et al., 2009) and also attenuates S^D^-induced cocaine seeking following both forced abstinence and extinction (Smith et al., 2010). Given such findings, it appears that orexin signaling may mediate both CS- and S^D^-driven reinstatement of drug seeking. In addition to MCH and orexin signaling, endogenous opioid peptides such as dynorphin have also been implicated in reward seeking (described in further detail below). It appears that neuropeptides may have a significant neuromodulatory role in controlling reward seeking and motivated behavior. Although, future studies will need to further elucidate whether the mechanisms driving these systems are conserved across drug classes and behavioral conditions.

##### Matrix metalloproteinases

Recently, neurobiological activity within the extracellular matrix (ECM), hypothesized to be the fourth component of the tetrapartite synapse (Smith et al., 2015), has become increasingly implicated in mediating structural plasticity that occurs in response to chronic exposure to drugs of abuse. Matrix metalloproteinases (MMPs), which are a family of zinc-containing endopeptidases, remodel the ECM and have been shown to mediate synaptogenesis, synaptic plasticity, and LTP (Ethell and Ethell, 2007). For example, studies have shown MMPs (particularly MMP-9) to be involved in dendritic remodeling of the dentate gyrus in response to kainate (KA)-induced excitotoxicity (Szklarczyk et al., 2002; Jourquin et al., 2003). In particular, systemic administration of KA is associated with increased expression of MMP-9 in the dentate gyrus of the HPC (Szklarczyk et al., 2002). Conversely, acute exposure to ethanol impairs spatial memory and is associated with decreased MMP-9 (but not MMP-2) activity in the HPC and PFC (Wright et al., 2003). Alterations in the expression and activity of MMPs within the mPFC as well as the NA have been implicated in drug seeking, where extinction of heroin self-administration is associated with a downregulation of MMPs in these regions. This neuroadaptation was partially restored following re-exposure to heroin-associated cues and acute pre-treatment with a broad-spectrum MMP inhibitor attenuated CS-induced reinstatement (Van den Oever et al., 2010). MMP activity has been shown to underlie changes in constitutive potentiation of glutamatergic synapses in the NAcore as well as changes in transient synaptic potentiation in response to conditioned stimuli paired with cocaine, nicotine, and heroin (Smith et al., 2014). Disruptions in MMP function and expression following both acute and chronic exposure to drugs appears to be conserved across drug classes and behavioral conditions. In fact, it has been suggested that these changes in MMPs might reflect a functional adaptation that resembles early developmental conditions in the brain (Smith et al., 2015), where the high expression of MMP-2 and MMP-9 during early development is significantly reduced over time (Ayoub et al., 2005). Given these findings, MMPs may be a potential therapeutic target for the treatment of substance use disorders. Nevertheless, the functional role of MMPs between CS- and S^D^-induced drug seeking is still unclear and more research is needed to elucidate whether MMP activity and its effects on drug seeking and synaptic plasticity is drug- and/or brain-region-specific between these two types of drug-associated stimuli.

##### Nitric oxide and endocannabinoids

Retrograde messengers such as nitric oxide (NO) and endocannabinoids (eCBs) are involved in critical signaling systems that mediate rapid changes in synaptic structure and function (see Regehr et al., 2009, for review). For example, inhibition of nitric oxide synthase (NOS) is associated with impairments in learning and memory, such as in spatial memory tasks (Estall et al., 1993; Yamada et al., 1995) as well as in olfactory memory tasks (Böhme et al., 1993). Recently, it has been suggested that long-term memories (such as drug-cue associations) can become labile upon retrieval and undergo re-consolidation processes that are susceptible to disruption (Hu and Schacher, 2015). In one study examining NO and the motivational properties of cocaine, inhibition of neuronal nitric oxide synthase (nNOS) activity was associated with decreased cocaine self-administration, extinction, and cocaine-primed reinstatement (although acquisition was unaffected) (Orsini et al., 2002). Smith, Scofield, Heinsbroek, Gipson and colleagues have recently shown that a small population of nNOS-expressing interneurons in the NAcore mediates glutamatergic plasticity of MSNs in response to cocaine-paired CSs and that this process is an mGluR5-dependent mechanism (Smith et al., 2017). In this study, pharmacological activation of mGluR5 as well as activation of designer Gq-coupled receptors on nNOS interneurons was associated with activation of nNOS in the absence of drug-associated CSs. Additionally, the degree of inactivation of nNOS interneurons was positively correlated with CS-induced reinstatement, and chemogenetic stimulation of nNOS interneurons was associated with an increase in MMP activity and AMPA currents in MSNs, both of which are known to drive CS-induced reinstatement (Smith et al., 2017). This study is the first to demonstrate that a small population of nNOS-expressing interneurons in the NA is capable of mediating transient plasticity induced by drug-associated cues. Considering nNOS activity is associated with increased drug seeking in CS-, S^D^-, and drug-prime models of reinstatement, NO signaling may constitute a neurobiological mechanism underlying relapse vulnerability.

Endocannabinoids comprise a family of endogenous retrograde messengers that are involved in a variety of physiological processes, such as pain-sensation, mood, hunger, learning, and memory (Aizpurua-Olaizola et al., 2017). The endocannabinoid system (ECS) also underlies the psychoactive effects of cannabis. The ECS mediates long-term depression (LTD) of synapses in regions such as the NA and HPC (Robbe et al., 2002; Chevaleyre and Castillo, 2003; Gerdeman and Lovinger, 2003) and dysregulations in this signaling system may underlie compulsive drug seeking. The cannabinoid receptor type 1 (CB_1_), which binds exogenous compounds such as Δ^9^-tetrahydrocannabinol (THC, the psychoactive constituent of cannabis) as well as endogenous cannabinoids such as anandamide and 2-arachidonoylglycerol (2-AG), has been shown to mediate both CS- and cocaine-induced reinstatement of drug seeking (De Vries et al., 2001). Similarly, CB_1_ receptors also mediate nicotine self-administration, where systemic antagonism of CB_1_ receptors following prolonged nicotine withdrawal decreased CS-induced nicotine seeking (Cohen et al., 2005). While these studies seem to suggest that blockade of CB_1_ receptors may suppress CS-induced drug seeking, one recent study found that inhibition of fatty-acid-amide-hydrolase (FAAH), the enzyme that degrades anandamide, decreased CS-induced nicotine reinstatement (which was reversed by administering a CB_1_ antagonist) (Forget et al., 2016). Therefore, induction and suppression of different aspects of the ECS may produce varying effects on drug-seeking behavior. It must be noted as well that alterations in the ECS have been shown to have long-lasting, transgenerational effects on cannabinoid, DA, and glutamate receptor genes along the mesolimbic pathway, where parental exposure to THC is associated with increased heroin seeking and decreased levels of GluN1 and GluN2B in the subsequent generation (Szutorisz et al., 2014). Whether this prenatal exposure can induce a heightened sensitivity to drug-associated cues later in life has yet to be fully elucidated. The ECS remains a putative target for treating substance abuse; however, our current understanding of the molecular mechanisms driving its effects on motivated behavior is still inadequate.

##### Neuronal ensembles and cue-induced reinstatement of drug seeking

Neuronal ensembles represent a subpopulation of functionally interconnected neurons that are collectively involved in specific computations. This concept was first developed by Donald Hebb in *The Organization of Behavior* (Hebb, 1949), where he postulated that neuronal cells could functionally assemble into “closed systems” and participate in various computations. Early work investigating neuronal ensembles in the NA found that cues associated with drug delivery produce activation of corticolimbic nuclei that converge onto and activate neuronal ensembles in the NA (Pennartz et al., 1994). More precisely, targeted inactivation of cocaine-activated neurons in the NA blocked locomotor sensitization specific to the cocaine context (Koya et al., 2009). Recent studies have examined this phenomenon in reinstatement paradigms, particularly in context-induced reinstatement of drug seeking. For example, Cruz et al. (2014) found that inactivation of NAshell but not NAcore neurons that were activated by a cocaine-associated context attenuated cocaine seeking behavior. Similarly, ensembles in the vmPFC are activated by a heroin-associated context and inhibition of these neurons attenuates context-induced (i.e., S^D^-induced) reinstatement of heroin seeking (Bossert et al., 2011). Neuronal ensembles in the OFC encoding heroin cues have also been shown to mediate cue-induced renewal of heroin seeking after a period of abstinence (Fanous et al., 2012). Interestingly, neurons encoding drug-cue associations are largely distinct from those encoding associations with natural rewards such as food (Carelli et al., 2000) and convergent lines of evidence seem to suggest that only a small proportion of cells (about 2–5%) encode cocaine memories (Mattson et al., 2008; Koya et al., 2009; Cameron and Carelli, 2012; Cruz et al., 2013). Recently, it has been suggested that rapid, transient synaptic potentiation of MSNs, which drives CS-induced drug-seeking behavior (Gipson et al., 2013a), may functionally expand the original ensemble due to glutamate spillover (Bobadilla et al., 2017). The underlying mechanism postulated here involves dysregulation of glutamate homeostasis following chronic drug exposure (Kalivas, 2009), which increases extrasynaptic levels of glutamate during reinstatement (Gipson et al., 2013a) and consequently activates NO and MMPs in an mGluR5-dependent manner. As described above, NO and MMP signaling mediate cue-triggered drug seeking and associated synaptic plasticity. As Bobadilla et al. (2017) describe, this transient “potentiation wave” may recruit additional neurons that reside adjacent to the neuronal ensemble as a consequence of this transient glutamate spillover, thus producing a robust behavioral response. In fact, 18% of the total recorded neurons in (Gipson et al., 2013a) expressed AMPA-to-NMDA (A/N) ratios that were at least two standard deviations above the mean A/N for cue-induced cocaine reinstatement. This contrasts with cue-induced sucrose seeking, where only 6% of the total recorded neurons expressed A/N ratios that were at least two standard deviations above the mean (Bobadilla et al., 2017). These findings potentially illustrate transient recruitment of additional neurons to the primary engram due to increased extracellular glutamate. We recommend that future studies examine this process further across drugs of abuse and between both CS- and S^D^-induced reinstatement paradigms to elucidate the cellular and molecular mechanisms that underlie the formation, persistence, and modulation of neuronal ensembles.

##### Other neurobiological targets involved in motivated behavior

Cue-type specificity is an important caveat underlying the neurobehavioral adaptations induced by drugs of abuse. Thus, future research investigating alternative neurobiological targets aimed at promoting drug abstinence and reducing relapse must take this into consideration. While not the primary focus of this review, the serotonin (5-HT) and norepinephrine (NE) neurotransmitter systems have also been extensively studied and are critically involved in both CS- and S^D^-induced drug seeking. For instance, the 5-HT_2A_ antagonist M100907 has been demonstrated to attenuate CS-induced cocaine reinstatement following extinction training (Nic Dhonnchadha et al., 2009). Intra-vmPFC injections of M100907 have also been demonstrated to inhibit CS-induced cocaine reinstatement (Pockros et al., 2011). Conversely, stimulation of 5-HT_2C_ with the agonist Ro60-0175 inhibits both CS- and S^D^-induced drug seeking (Burbassi and Cervo, 2008; Fletcher et al., 2008). In addition to 5-HT, NE receptors (particularly the α_2_ receptor) have also been demonstrated in rodents to mediate both drug- and CS-induced reinstatement, although these findings are mixed in nonhuman primates (for review, see Zaniewska et al., 2015).

One recent study examining the role of voltage-gated calcium channels (VGCC) in the NA found that selective antagonism of the α_2_δ-1 VGCC subunit with gabapentin attenuated cocaine-primed (but not CS-induced) reinstatement following cocaine self-administration, where cocaine infusions were paired with discrete light and tone cues (Spencer et al., 2014). As mentioned previously, certain neuropeptide systems may be potential neurobiological targets aimed at reducing drug seeking. For example, selective activation of the kappa opioid receptor (KOR) by dynorphin along the mesolimbic pathway inhibits VTA and NA DA release (Margolis et al., 2003). Recent evidence suggests that the dynorphin/KOR system maintains drug seeking since both ethanol (Walker and Koob, 2008) and nicotine self-administration (Galeote et al., 2009) depend on dynorphin/KOR signaling. Corticotropin-releasing factor (CRF) is another neuropeptide that has been implicated in drug seeking, particularly in stress-induced reinstatement of drug seeking, where a footshock can serve as discrete conditioned stimulus that reinstates drug seeking (Koob and Le Moal, 2005). While manipulating these neuropeptide systems may be efficacious in attenuating drug seeking, it is not entirely clear what specific role these substrates play in CS- vs. S^D^-induced drug seeking.

Another potential neurobiological target is the molecular clock. Many studies have implicated circadian rhythms as important mediators of drug seeking (Falcón and McClung, 2009). As such, manipulating transcriptional regulators of this system may be effective in suppressing drug seeking. Indeed, diurnal and circadian patterns of behavior are heavily reliant on environmental stimuli (e.g., zeitgebers), so careful consideration should be taken regarding how particular cue types impact this molecular system. Recently, sex differences in both clinical and pre-clinical settings have been observed in the training, maintenance, withdrawal, and relapse of drug use (Becker and Koob, 2016). Notably, gonadal hormones appear to play a significant role in motivated behavior and may be a potential neurobiological target in the treatment of substance abuse. For instance, one study found that cocaine-seeking following an extended period of withdrawal was enhanced during estrus in female rats relative to non-estrus females or males (Kerstetter et al., 2008). However, how these hormones specifically impact sensitivity to conditioned or discriminative drug-associated cues has yet to be determined.

## Discussion

Much preclinical research has used contingent CSs as conditioned reinforcers to produce drug seeking behavior (See, 2002), and the construct validity of this reinstatement model must be taken as a caveat to interpreting the translational reach of the neurobehavioral mechanisms underlying this motivated behavior (Epstein et al., 2006). The contingent CS-induced reinstatement model, however, has led to pharmacotherapeutic developments that have shown some translational relevance in reducing drug relapse and craving in human clinical studies across different drug classes (Mardikian et al., 2007; Knackstedt et al., 2009; Gray et al., 2010; Berk et al., 2013; McClure et al., 2014, 2015). Preclinically, similar neuroadaptations in glutamatergic signaling within the mesocorticolimbic pathway have been reported with different drugs of abuse including nicotine, cocaine, and heroin (LaLumiere and Kalivas, 2008; Shen et al., 2011; Gipson et al., 2013a,b; Scofield et al., 2016). However, an appreciable amount of neurobiological heterogeneity exists even within contingent CS-induced drug seeking, where corticothalamic and corticostriatal neuronal projections from the PFC have opposing functional roles in controlling reward-seeking behavior (Otis et al., 2017).

Compared to the contingent CS-induced reinstatement model, the neurobehavioral mechanisms that underlie drug seeking induced by non-contingent S^D^s or OSs is sorely understudied. Additionally, what we do know about these stimuli has been derived from models using context as the S^D^. These non-contingent modulatory stimuli occur often in the lives of substance abusers and can lead to increased drug craving and relapse in humans (Childress et al., 1993). Clinical study regarding the effects of drug cues is almost exclusively done with models that utilize non-contingent presentation of drug-associated stimuli, including stimulus-induced craving and attentional bias (Sinha and Li, 2007; Field and Cox, 2008). Thus, using similar methods in preclinical models, like OS- and S^D^-induced drug seeking, may help to identify more clinically relevant neurobehavioral mechanisms underlying substance use disorders. Likewise, increased use of contingent CS models in human clinical studies may help to validate existing preclinical models. Together these approaches may aid in forging a better bridge between clinical and preclinical research on cue effects in substance abuse, helping to identify future therapeutics.

As mentioned above, a potential caveat of previous models of cue effects in drug seeking is the lack of specificity. Most prior research using either contingent CSs or non-contingent S^D^s to study cue effects on drug seeking has been done in isolation of other reinforcers. If an attempt to demonstrate specificity of either treatment effects or stimulus effects is made, it is typically done through a separate group of drug-naïve animals trained to respond solely for a natural reinforcer, like food. However, chronic exposure to drugs of abuse results in dramatic changes within the brain, affecting reward, learning, memory, and decision-making processes, and how a reward-associated stimulus interacts with a drug-naïve system is very different than in a drug-exposed system (Hyman et al., 2006). Thus, it is important for future research to dissociate drug-associated neurobehavioral processes from those associated with natural reinforcers *within* a system that has been chronically exposed to drugs of abuse to better aid in the future discovery of more specific behavioral and pharmacological therapies for substance use disorders. Toward this goal, there have been a few attempts to utilize preclinical models that allow for direct comparison of stimulus control of drug seeking by drug-associated cues vs. control of food seeking by food-associated cues within animals chronically exposed to drugs of abuse (Weiss et al., 2003; Kearns and Weiss, 2007; Weiss et al., 2007; Lombas et al., 2008; McCuddy et al., 2014). Although these models demonstrate clearly specific control of drug and food seeking by their associated stimuli, the neurobehavioral mechanisms that underlie this differential control are unknown. Only one study to date has evaluated multimodal reinstatement, illustrating specific stimulus control over food and drug taking within an animal (Batten and Beckmann, 2017). Future research into the neurobehavioral processes that govern differential stimulus control of seeking drug vs. natural reinforcers will help in identifying more specific neurobehavioral targets for future therapies to treat reward-related pathologies.

It is important to note that studies examining the neurobiological mechanisms underlying contextual S^D^s in comparison to CSs don't consider that contextual stimuli are spatially-variable, multimodal stimuli and may incorporate multiple sensory systems and themselves engage distinct neural circuits (e.g., hippocampal nuclei) that may not be specifically related to their function as S^D^s within the context-induced drug seeking procedure. Thus, future studies should employ a single stimulus (e.g., a light) to serve as both a contingent conditioned reinforcer as well as a non-contingent S^D^ in order to dissociate the neurobehavioral processes underlying the functional differences between these two types of stimuli.

## Conclusions

We propose that the models discussed herein are useful in determining the differential neurobiological substrates underlying various types of motivated behavior and that care should be taken to better dissociate the roles of different types of cues in models of relapse. As we have attempted to highlight in this review, there is a tendency in the substance use disorder literature (both clinical and preclinical) to posit the stimulus control of drug-associated behavior as the product of a single, unitary Pavlovian process. However, stimulus-induced drug seeking and associated craving, as highlighted herein, can manifest through various neurobehavioral mechanisms that recruit differential neural circuitry, including conditioned reinforcement and modulatory effects on both operant and Pavlovian relations via S^D^s and OSs, respectively. Thus, stimulus-induced drug seeking and relapse is not a unitary process and it is imperative to pay closer attention to the functional role of drug-associated stimuli during the development of behavioral and pharmaceutical interventions. Contingent CS models of reinstatement have yielded important and informative results that have led to translationally valuable advances in the development of pharmacotherapeutics to reduce relapse vulnerability. Regardless, our knowledge of their modulatory counterparts (e.g., S^D^ and OS) is comparatively infinitesimal. Thus, there is much room for improvement as relapse rates remain high and pharmacotherapeutic advancements that have emerged from these preclinical models have somewhat checkered success in the clinic (Knackstedt et al., 2009; LaRowe et al., 2013; McClure et al., 2014). Future research should attempt to directly dissociate the neurobehavioral mechanisms underlying the stimulus control of drug seeking associated with CS, S^D^, and OS models, as behavioral and pharmacotherapeutic development for intervention of drug relapse may be improved by systematically examining these functionally distinct paths to drug seeking. As highlighted throughout this review, S^D^ reinstatement models of drug relapse almost exclusively utilize contextual cues as the S^D^. Thus, future research should employ discrete (as opposed to contextual) discriminative cues to further elucidate the neurobehavioral mechanisms underlying these distinct stimuli. Moreover, polydrug abuse is a typical feature of substance use disorders, where abuse of multiple drugs can hinder successful treatment outcomes (Wang L. et al., 2017). Specifically, primary drug use, which is defined as the main substance reported at the time of admission into a treatment program (Mattson et al., 2017), is modulated by use of secondary substances. No studies to date have thoroughly examined how primary drug cues influence secondary drug seeking and vice versa. Such research would reveal novel insights into how complex drug-cue associations modulate drug seeking behavior in individuals who engage in polydrug use. Finally, future research should also incorporate stimulus control of alternative sources of reinforcement, such as in a multiple schedule, to better isolate drug-associated stimulus control in models of chronic drug exposure. There are numerous and varied roads to relapse. Therefore, both behavioral and pharmacotherapeutic interventions must be tailored to the individual to promote better drug cessation outcomes.

## Author contributions

MN, JB, and CG designed and wrote the paper. ST and MFO edited and contributed to sections of the paper.

### Conflict of interest statement

The authors declare that the research was conducted in the absence of any commercial or financial relationships that could be construed as a potential conflict of interest.
